# Myasthenia Gravis in a Child With Schimke Immuno‐Osseous Dysplasia: A Case Report

**DOI:** 10.1002/ccr3.71604

**Published:** 2025-12-07

**Authors:** Mohamed S. Al Riyami, Intisar Al Alawi, Maisa Al Riyami, Badria Al Gaithi, Suliman Al Saidi, Ibtisam Al Shuaili, Naifain Al Kalbani, Nabil Al Maki

**Affiliations:** ^1^ Pediatric Nephrology Unit, Department of Child Health Royal Hospital Muscat Oman; ^2^ National Genetic Center, Ministry of Health Muscat Oman; ^3^ Department of Radiology Royal Hospital Muscat Oman; ^4^ Pediatric Neurology Unit, Department of Child Health Royal Hospital Muscat Oman

**Keywords:** autoimmunity, end‐stage renal failure, myasthenia gravis SMARCAL1 gene mutation, Schimke immune‐osseous dysplasia (SIOD), steroid‐resistant nephrotic syndrome

## Abstract

We report a rare association between Schimke immune‐osseous dysplasia and myasthenia gravis. Clinicians should be aware of potential autoimmune neuromuscular complications in SIOD, as early recognition and tailored immunosuppression may improve prognosis.

## 1 Introduction

Schimke immune‐osseous dysplasia (SIOD) is an autosomal recessive disorder characterized by impaired cellular immunity, lymphopenia, spondyloepiphyseal dysplasia, and growth retardation. It was first described by Robert Neil Schimke in 1971 [1]. It results from biallelic mutations in the SMARCAL1 gene, identified by Dr. Cornelius Boerkel in 2002, who also established the genotype–phenotype correlation associated with this condition [2].

The SMARCAL1 protein features a helicase domain responsible for binding and unwinding double‐stranded DNA, contributing to the overall stabilization of DNA molecules. While the helicase functions are understood, the exact mechanisms through which SMARCAL1 dysfunction leads to the specific phenotypic manifestations of SIOD remain poorly defined [1, 3]. The helicase domain primarily comprises two subdomains, including an ATPase domain, where most identified mutations in SIOD patients occur [1].

SIOD is considered a rare disorder, with an estimated prevalence of 1 in 1–3 million individuals. However, data from the PodoNet registry and American registries indicate that approximately 1% of patients diagnosed with steroid‐resistant nephrotic syndrome carry mutations in the SMARCAL1 gene [3].

Clinically, patients with SIOD present with distinctive features, including disproportionate short stature characterized by a short neck and trunk, lumbar lordosis, and a protruding abdomen. Pigmented macules are often present on the trunk, and facial features typically include a triangular face, broad nasal bridge with a bulbous nasal tip, microdontia, hypodontia, and malformations of both deciduous and permanent molars [4].

While most individuals with SIOD demonstrate normal intellectual functioning, some may experience cognitive impairment or mild developmental delays following cerebral insults [5]. Approximately 50% of SIOD patients are susceptible to recurrent infections due to T‐cell deficiency, which is marked by decreased CD4+ lymphocyte levels and abnormal immunoglobulin concentrations. This T‐cell dysfunction also elevates the risk for lymphoproliferative disorders, including non‐Hodgkin lymphoma [4].

Additionally, autoimmune disorders are prevalent in SIOD, encompassing conditions such as autoimmune thrombocytopenia, autoimmune anemia, autoimmune bowel disease, and central nervous system autoimmune disorders, including acute disseminated encephalomyelitis [6].

The increasing application of gene panel screening for patients presenting with steroid‐resistant nephrotic syndrome and proteinuria has led to the earlier identification of SIOD cases in clinical settings [3]. To date, no cases of SIOD complicated by myasthenia gravis (MG) have been reported in the literature. This case is therefore unique in illustrating a novel autoimmune neuromuscular manifestation of SIOD, expanding the known clinical spectrum of the disease. By documenting this association, we aim to raise awareness among clinicians of the need to consider autoimmune comorbidities in SIOD and to contribute to the limited data on genotype–phenotype and immune dysfunction in this ultrarare disorder.

## 2 Case History/Examination

A 10‐year‐old girl was first evaluated at the Royal Hospital at the age of 4 years following a referral from a local hospital due to incidental proteinuria detected during an assessment for body itching and dryness. At that time, her blood pressure, renal function, cholesterol levels, and serum albumin were within normal ranges. A kidney biopsy performed after 4 months of presentation revealed features consistent with focal segmental glomerulosclerosis (FSGS), and genetic testing for NPHSII was negative. Both hearing and ophthalmological evaluations were unremarkable.

The patient lost follow‐up and then presented after 8 months with features consistent with nephrotic syndrome. Notably, hypertension and hypothyroidism were also identified. Initial treatment commenced with prednisolone at a dosage of 60 mg/m^2^/day for 6 weeks, followed by alternate‐day dosing for 4 weeks, with a subsequent gradual tapering. However, the patient did not respond to this therapy. Tacrolimus was then initiated but was discontinued after 6 months due to unresponsiveness. She was concurrently prescribed Enalapril and Amlodipine to manage proteinuria and hypertension, thyroxine for hypothyroidism, and Atorvastatin for hypercholesterolemia.

Family history revealed three healthy siblings, with parents who are second‐degree consanguineous, and no known family history of renal disease. During follow‐up, the patient exhibited a progressive decline in renal function, ultimately reaching end‐stage renal disease by age 9, leading to the initiation of automated peritoneal dialysis (APD).

Additionally, the patient presented with persistent lymphopenia and experienced multiple episodes of otitis media related to upper respiratory tract infections, as well as a single episode of shingles that required hospitalization for intravenous acyclovir. There was no reported history of recurrent pneumonia, abscesses, or severe infections.

Physical examination revealed disproportionate short stature and subtle dysmorphic features, including a triangular facial shape, bulbous nasal tip, short neck and trunk, lumbar lordosis, hyperpigmented skin macules on the neck and trunk, and a protruding abdomen.

Whole exome sequencing was conducted, identifying a homozygous variant of uncertain significance in the SMARCAL1 gene (c.2547G>T p.(Lys849Asn)).

## 3 Differential Diagnosis, Investigations, and Treatment

In February 2024, the patient was admitted with a 1‐month history of generalized weakness exhibiting diurnal variation, particularly worsening in the evening. She reported easy fatigability during short walks and difficulties when rising from a seated position. Bulbar symptoms included dysphagia, decreased vocal tone, and swallowing difficulties, without any gastrointestinal or urological complaints.

During examination, the patient was alert and oriented, noted to have bilateral ptosis, restricted facial movements, and bulbar symptoms. Muscle weakness was exacerbated by repetitive movements. The initial differential diagnosis considered myasthenia gravis or a metabolic myopathy. Laboratory tests revealed acetylcholine receptor antibody levels of 0.411 nmol/L, categorized as borderline high. Neurophysiological evaluation via repetitive nerve stimulation test (RNST) performed in the right ulnar nerve and right hypothenar muscles shows a decrement response > 10% suggestive of a likely diagnosis of myasthenia gravis. Chest MRI did not reveal any significant thymic mass.

**FIGURE 1:**
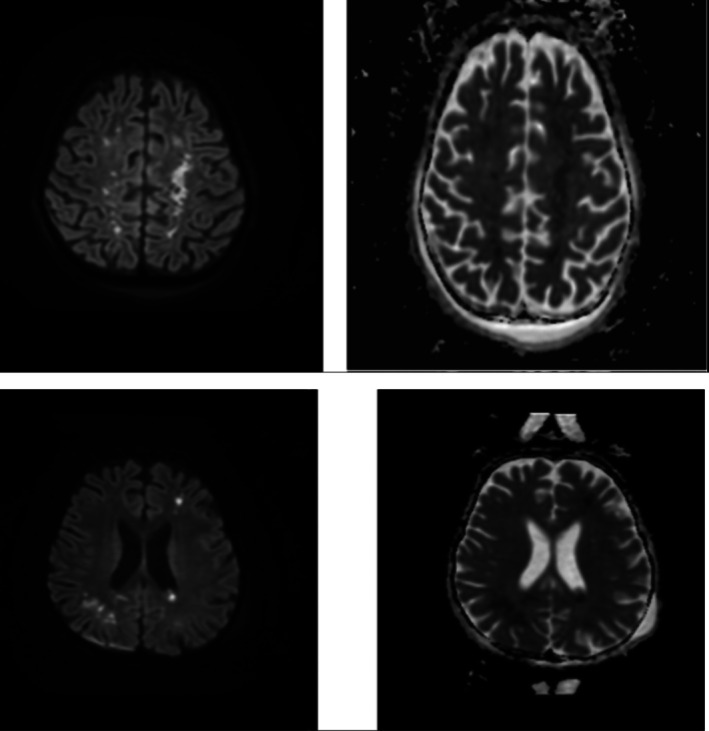
MRI brain showed diffusion weighted images and ADC map show multiple focal areas of diffusion restriction in bilateral centrum semiovale and subcortical white of frontal and parietal lobes bilaterally.

The patient was initially treated with a low dose of pyridostigmine, which was subsequently increased to 5 mg/kg/day administered four times daily. However, her condition worsened, necessitating transfer to the pediatric intensive care unit (PICU) due to flaccid paralysis and respiratory failure requiring mechanical ventilation. She received treatment for a myasthenic crisis, including plasma exchange, pulse methylprednisolone, and intravenous immunoglobulin. Despite these interventions, her condition continued to deteriorate, resulting in dysautonomia and profound encephalopathy characterized by minimal responsiveness (Figure 1).

Electroencephalogram (EEG) findings showed bilateral mild to moderate slowing of background activity, with no sleep markers observed. Neurological examination revealed upper motor neuron signs in both upper and lower limbs, including exaggerated reflexes and spasticity, more pronounced on the right side. An MRI of the brain demonstrated abnormal white matter hyperintensities consistent with microinfarcts, suggestive of acute small vessel disease, while MRA confirmed normal vascular anatomy (Figure 1).

**FIGURE 2:**
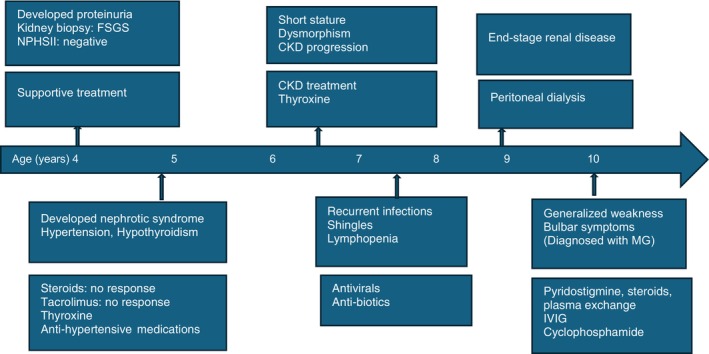
Timeline of evolution of clinical manifestations and treatment given.

The patient remained mechanically ventilated for 3 weeks, continuing to exhibit symptoms of myasthenia gravis and limb spasticity. A multidisciplinary team meeting resulted in the decision to initiate cyclophosphamide pulse therapy and to consider tracheostomy if mechanical ventilation was still required. A single dose of cyclophosphamide was given. Two weeks later, the patient showed improvement in her condition, allowing for successful weaning from mechanical ventilation and extubation. Postextubation, she exhibited greater weakness in her right upper and lower limbs compared to the left (Figure 2).

## 4 Outcome and Follow‐Up

She was subsequently transferred to the pediatric ward for physiotherapy and discharged home on a tapering regimen of prednisolone, pyridostigmine, medications for her chronic kidney disease and thyroid hormone replacement. In subsequent outpatient evaluations by the pediatric neurology team, the prednisolone dosage was reduced, and pyridostigmine was discontinued, leading to a recurrence of symptoms. This prompted readmission and reinitiation of treatment with steroids and pyridostigmine, along with the introduction of a small dose of mycophenolate mofetil as a steroid‐sparing agent. The patient's condition improved under this revised treatment plan.

## 5 Discussion

This case report presents a child diagnosed with SIOD, who initially exhibited proteinuria due to FSGS at the age of 4. The condition did not respond to immunosuppressive therapy, leading to the development of chronic kidney disease and the initiation of APD. Subsequently, the patient developed symptoms indicative of myasthenia gravis (MG), which progressed to a myasthenic crisis characterized by bulbar symptoms and respiratory weakness. To our knowledge, this represents the first documented case of myasthenia gravis in a patient with SIOD. Conventional treatments, including oral prednisolone, plasma exchange, and intravenous immunoglobulin, were ineffective, and the patient received a single dose of intravenous cyclophosphamide. Furthermore, she suffered an ischemic brain infarction as a result of vascular complications.

Renal disease in SIOD typically manifests before the age of 12 and can progress to end‐stage renal disease (ESRD) within 1 to 11 years. Both early‐onset nephropathy (by age 5) and juvenile‐onset nephropathy (postage 10) have been reported, often diagnosed in conjunction with growth deficiency [3]. The predominant renal pathology associated with SIOD is FSGS [4].

Our patient exhibited persistent lymphopenia, recurrent episodes of otitis media, and a single episode of shingles, with no history of severe infections. Approximately 80% of individuals with SIOD experience T cell deficiency, primarily of the memory phenotype, which is associated with diminished thymic production linked to a lack of interleukin‐7 receptor alpha, impairing T cell development [7]. Although B cell counts are generally normal, hypogammaglobulinemia is common, often necessitating immunoglobulin replacement therapy [4]. Additionally, around 38% of affected individuals present with neutropenia, increasing the risk of opportunistic infections, such as *Pneumocystis jirovecii* pneumonia, which can lead to severe morbidity and mortality [2].

Children with SIOD frequently encounter various autoimmune disorders, including autoimmune thrombocytopenia, anemia, and central nervous system complications [6]. The mutation in the SMARCAL1 gene, which is essential for DNA stabilization, contributes to immunodeficiency and heightened susceptibility to infections and autoimmune diseases, likely due to disturbances in T cell regulation [8]. In this context, autoimmune MG is characterized by neuromuscular transmission impairment due to antibodies against acetylcholine receptors (AChR) or muscle‐specific kinase (MuSK) [9, 10]. The association between SIOD and MG remains speculative, but plausible mechanisms include T‐cell dysregulation due to impaired thymic output and IL‐7Rα deficiency in SIOD, which could predispose to loss of self‐tolerance and autoantibody generation [7]. Additionally, molecular mimicry following recurrent infections in immunodeficient patients may further trigger autoimmunity at the neuromuscular junction [11, 12] Our patient demonstrated borderline AChR antibody levels and exhibited typical MG symptoms, such as bilateral ptosis and respiratory failure, necessitating mechanical ventilation. Neurophysiological assessments utilizing repetitive nerve stimulation (RNS) in our patient provide evidence supporting the diagnosis of MG. Repetitive nerve stimulation and single‐fiber electromyography (SFEMG) are established screening methods for myasthenia gravis. The sensitivity of SFEMG is approximately 95%; however, it necessitates voluntary muscle activation, which can be challenging or inconsistent in young children [13].

Initial treatment with pyridostigmine proved ineffective, leading to the incorporation of methylprednisolone, plasma exchange, intravenous immunoglobulin and received one dose of Cyclophosphamide. Following recurrent symptoms, mycophenolate mofetil was introduced as a steroid‐sparing agent. While no specific guidelines exist for the treatment of MG in pediatric patients, prednisolone remains the primary therapy, with alternatives like azathioprine and rituximab available for cases that do not respond adequately [14]. Plasma exchange has shown greater efficacy than intravenous immunoglobulin for managing acute exacerbations [13].

Thymectomy was unnecessary for our patient, given the absence of thymoma on chest X‐ray and MRI, as well as the borderline elevation of AChR antibody levels. Thymectomy is generally recommended for patients with generalized juvenile myasthenia gravis (JMG) who are positive for acetylcholine receptor (AChR) antibodies, although its therapeutic benefit in ocular JMG or seronegative disease remains uncertain [13]. The management of JMG is largely determined by antibody status. AChR‐positive cases typically respond to corticosteroid therapy and may derive additional benefit from thymectomy, whereas seronegative or muscle‐specific kinase (MuSK)‐positive subtypes often require alternative immunosuppressive agents such as rituximab [13]. In the present case, the combination of borderline AChR antibody positivity and absence of thymoma supported a conservative, nonsurgical approach, in alignment with published clinical guidelines [15]. This case highlights the importance of individualized therapeutic strategies in syndromic JMG, particularly when concomitant immunodeficiency and renal disease complicate conventional treatment algorithms. Furthermore, immunosuppressive therapies administered for SIOD, including corticosteroids and tacrolimus, may have influenced the clinical course of MG, potentially attenuating early manifestations or altering treatment responsiveness. Conversely, intensive immunosuppressive regimens commonly used in MG, such as high‐dose corticosteroids or cyclophosphamide, carry the risk of aggravating SIOD‐associated immunodeficiency, thereby necessitating a careful therapeutic balance.

Other inherited syndromes, such as von Hippel–Lindau (VHL) disease, have also been associated with MG, including both thymoma‐associated, antibody‐positive presentations and nonthymoma cases, suggesting that genetic susceptibility may contribute to B‐cell–mediated autoimmunity [16, 17, 18].

In Patients with MG are at increased risk of concomitant autoimmune diseases, particularly autoimmune thyroid disease (ATD), systemic lupus erythematosus (SLE), rheumatoid arthritis (RA), dermato‐/polymyositis, and Addison's disease. Autoimmune overlap occurs in 13%–22% of MG patients, most frequently in women and in early‐onset MG (EOMG). ATD is the most common comorbidity, observed in ~10% of cases. Genetic predisposition, especially HLA haplotypes such as HLA‐B8‐DR3, contributes to both MG susceptibility and associated autoimmune conditions [19].

During her stay in the PICU, our patient also developed encephalopathy and bilateral weakness secondary to cerebral infarction, with more pronounced involvement on the right side compared to the left. Follow‐up assessments indicate that her weakness has improved. Neurological manifestations in patients with SIOD include early‐onset cerebral ischemic attacks, migraine‐type headaches, optic neuropathy, seizures, intellectual disability, and behavioral changes [20]. These clinical features are thought to result from underlying pathological mechanisms such as progressive atherosclerosis and intrinsic neurovascular defects. The potential etiopathology of these symptoms may be associated with mutations in the SMARCAL1 gene, which is highly expressed in the developing brain of both adult mice and humans, including neural precursor and neuronal lineage cells [5].

Jakub Zieg et al. reported three patients with SIOD who developed neurological symptoms. One patient experienced triplegia with expressive aphasia due to recurrent ischemic attacks, while two other patients suffered from recurrent transient ischemic attacks, strokes, and headaches. Over time, the duration of these attacks increased, and the symptoms became more severe [21].

Andrzej Badeński et al. reported a case of a 14‐year‐old girl with SIOD who presented with recurrent neurological symptoms, including migraine‐like headaches, diplopia, and seizures. These symptoms may be attributable to cerebral vasoconstriction syndrome (RCVS), which has been described as a potentially novel mechanism for cerebrovascular complications and headaches in SIOD [14].

The SMARCAL1 variant identified in our patient (c.2547G>T; p.Lys849Asn) is classified as a variant of uncertain significance (VUS) according to ACMG criteria, as no functional assays or segregation studies were available. To our knowledge, this variant is not listed in gnomAD or previously reported in association with SIOD. While the clinical phenotype was strongly consistent with SIOD, we emphasize that the genetic finding remains a VUS, and the diagnosis rests on the integration of clinical, radiological, and immunological features rather than definitive molecular confirmation. Nonetheless, the clinical presentation supports a diagnosis of SIOD. Severe early‐onset phenotypes are typically associated with truncating variants of SMARCAL1, which result in absent protein production, while compound heterozygous missense variants may lead to milder, nonrenal phenotypes due to the presence of an unstable protein [3]. Definitive genotype–phenotype correlations remain to be elucidated, and the relationship between the type or severity of disease‐causing variants and renal impairment has yet to be clearly defined. The study by Bertulli et al. describes two brothers with SIOD carrying the same homozygous missense variant (p.Arg561His) in the SMARCAL1 gene, but with differing clinical severities. The first patient experienced severe growth retardation, developmental delays, and multiple organ malformations, while the second patient presented with multicystic dysplastic kidney (MCDK) and an aggressive type of congenital mesoblastic nephroma (CMN). Despite the expectation that missense variants would lead to milder phenotypes, both siblings showed severe symptoms, challenging the genotype–phenotype correlation. Notably, they also exhibited combined immunodeficiency with impaired NK and B cell functions [22]. These findings suggest that clinical outcomes in SIOD cannot be strictly predicted by genetic variants, highlighting the need for tailored treatment approaches and further research into the disease's complexities.

## 6 Conclusion

This case illustrates a previously unreported association between Schimke immune‐osseous dysplasia (SIOD) and myasthenia gravis (MG). It expands the clinical spectrum of SIOD and highlights the importance of considering autoimmune neuromuscular manifestations in affected patients. Early recognition and multidisciplinary management are essential to improve outcomes in this ultra‐rare disorder.

## Author Contributions

**Mohamed S. Al Riyami:** conceptualization, data curation, formal analysis, supervision, writing – original draft, writing – review and editing. **Intisar Al Alawi:** methodology, writing – original draft, writing – review and editing. **Maisa Al Riyami:** data curation, writing – original draft. **Badria Al Gaithi:** formal analysis, resources. **Suliman Al Saidi:** formal analysis, methodology, writing – review and editing. **Ibtisam Al Shuaili:** resources, writing – original draft. **Naifain Al Kalbani:** resources, visualization, writing – review and editing. **Nabil Al Maki:** resources, writing – review and editing.

## Funding

The authors have nothing to report.

## Consent

Written informed consent was obtained from the patient for the publication of this case report and any associated images. A copy of the signed consent form is available for inspection by the Editor of the journal upon request.

## Conflicts of Interest

The authors declare no conflicts of interest.

## Data Availability

Data sharing is not applicable to this study, as no new data were generated or analyzed.
